# Determining the (A)symmetric Role of Business–Consumer Confidence in Outward–Inward Tourism in Russia: A Competitiveness Perspective

**DOI:** 10.1007/s42943-023-00077-z

**Published:** 2023-06-05

**Authors:** Uju Violet Alola, Darya Baeva, Andrew Adewale Alola

**Affiliations:** 1 Department of Tourism Guidance, Faculty of Economics, Administrative and Social Science, Istanbul Gelisim University, Istanbul, Turkey; 2 Department of Economics and Finance, South Ural State University, Chelyabinsk, Russian Federation; 3 CREDS-Centre for Research on Digitalization and Sustainability, Inland Norway University of Applied Sciences, 2418 Elverum, Norway; 4 Faculty of Economics, Administrative and Social Sciences, Nisantasi University, Istanbul, Turkey

**Keywords:** Tourism competitiveness, Market confidence, Consumer price index, (Non)linearity, Frequency domain causality, Russia, C22, L83, O52

## Abstract

Tourism activities in Russia, prior to the coronavirus pandemic and the Ukraine–Russia conflict, reportedly recorded a desirable boost, thus prompting the formulation of the country’s new Tourism Development Strategy for 2035. Considering this observation, the current study examines the determinants of the Russia’s inbound and outbound tourism, but especially from the perspective of linear and nonlinearity role of market—business and consumer—confidence over the quarterly period 2010Q1–2020Q4. The result shows that negative and positive shock in business confidence and consumer price index positively affects international arrivals to Russia in the short and long run, but with a negative shock in business confidence showing a larger effect. A shock (irrespective of the direction) in the consumer leading indicator leads to decline in international arrivals in the short and long run. We also found that a shock (mostly negative) in the consumer confidence reduces the numbers of Russian travellers to foreign tourist destinations in the short- and long-term period. However, a negative shock in business confidence encourages Russians to embark on touristic visit to destinations, while there is no significant effect arising from the shock in the consumer price index. Moreover, for symmetric evidence, there is a significant two-way Granger causality from BCI to outbound tourism, and from CLI to outbound tourism especially in the medium term, while a one-way causality exists from CPI to outbound tourism in the short and medium term.

## Introduction

With tourism activities accounting for a significant economic and development impact (United Nations World Tourism Organization, 2021), the performance of this industry across the European region and especially in Russia is also vital. For instance, international and domestic tourism arrivals to Russia in 2018 (just before the coronavirus pandemic) increased by 0.7% and 16.2%, respectively, as compared to 2017 and reportedly generated 540,500 employments in 2017 (Organization for Economic Co-operation & Development, 2021). According to the said report, the economic aspects that are increasingly promoting tourism activities within Russia include traditional tourism, education, sport (such as skiing), cruises, business, medical, and many others. To achieve expansion and sustainable industry, a new Tourism Development Strategy (TDS) is designed to meet the outlined 2035 target. However, like every other economic sector, tourism activities in Russia are not immune to the deterministic factors that are capable for navigating the industry pathway. For instance, the social, economic, political stability, security, institutional factors, and many more, as applicable to several destination countries (Akadiri et al., 2020; Alola et al., 2021; Athari et al., 2021), are largely applicable to Russia. Interestingly, the outbound tourism in most cases could be somewhat affected by some of the aforementioned factors, which have been widely discussed in the extant literature.

Considering the above motivation, the design of the current study is in the direction of examining the determinants of inbound and outbound tourism in the Russian Federation from the perspective of business and consumer confidence. While employing a quarterly dataset over the quarterly period 2010Q1–2020Q4, the novel objective of the study is tailored towards revealing evidence of linear and nonlinearity relationship between business confidence and tourism aspects (inbound and outbound tourism) and consumer confidence (consumer leading indicator as a proxy) and the tourism aspects. Additionally, the (non)linearity link between inflation indicator (consumer price index as a proxy) and the tourism aspects is also considered for examination. Moreover, employing a frequency domain Granger causality approach to reveal the causative relationships that provides frequency inference further adds to the relevance and novelty of the study. By considering the case of Russia, which has received limited attention in the tourism literature, the current study, in addition to achieving the aforementioned objectives in such a novel dimension, is expected to make significant contribution to the literature. Moreover, given the potential to improve competitiveness in the industry, similar to other industry like the banking sector (Oduro et al., 2022), the result from this investigation should guide policymakers and practitioners to achieve a competitive drive.

To provide a coherent structure, the outline of the study is in the order of literature (in Sect. "Literature Review"), description of the dataset and empirical analysis (Sect. "Dataset and Method"), discussion of the results (Sect. "Discussion of Findings"), and the conclusion of the study with policy formulation (Sect. "Conclusion and Policy-Related Dimension").

## Literature Review

The presentation of empirical literature in this section is in two dimensions: (1) the discussion by empirical studies of the nexus between business confidence and the measure of tourism aspects such as inward and outward tourism and (2) the discussion by empirical studies of the nexus between consumer leading indicators (such as consumer confidence) and the aspects of tourism development.

### Business Confidence and Tourism Aspects

Several literature works have acknowledged that confidence measures are very vital in predicting the macro variables in tourism; however, there exist handful of such studies (Ang, 2010; Wu & Wu, 2021). Construction and analysis of business confidence index is essential in predicting economic development, that is to say that it contributes to the knowledge of the valuable insights into business people’s psychological and social circumstances surrounding the business environment and these improve the extent to which investment is reliable (Khan & Upadhayaya, 2020). The United Nations has mapped out the European Commission’s Economic Sentiment Index (ESI) as a vital instrument for investigating business confidence indicators (European Commission, 2023). For this reason, the measurement of business confidence needs to be measured accurately. The use of the business confidence index, especially during economic downturns and world shutdown in the case of the pandemic, has received little attention.

However, several researchers investigated international business travels and its effect on business openness and business expansion (Tan & Tsui, 2017; Tsui & Fung, 2016). Additionally, the studies of Tsui et al. (2019) and Cortse-jimenez and Blake (2011) examine the determinants of international business arrivals, and Turner and Witt (2001) are of the opinion that several factors influence business tourism demand (retail sales, new private car and domestic loans) when they examined a period of 19 years spanning from 1978 to 1997 five countries. An in-depth knowledge to find out if there is any relationship between tourist demand when it is positive and when it is negative is vital for decision forecasting (Lee et al., 2011; Sheldon & Dwyer, 2010). However, some researchers have opined that the tourism expenditure demand during bad times and good time differs (Bronner & de Hong, 2016; Smeral & Song, 2015; Smeral, 2010, 2012). The tourism demand in the economic boom is not asymmetric to the period of economic recession. Tourists exhibit different behaviour during economic crisis (Cellini & Cuccia, 2015). The knowledge of this exploration is of great concern for policymakers. Moreover, openness to business, availability of holiday tourism, and country’s financial stability are identified as vital economic determinants that explain the reason people travel to destinations (Kulendran & Wilson, 2000; Kulendran & Witt, 2003).

A more recent study examined how tourist arrival in Greece is influenced by BC both in the country of origin and in the destination country by using a time series approach concluding that BC was not found to be a major determinant (Chatziantoniou et al., 2016). On the other hand, some researchers highlighted the four factors (distance from other countries, economic policy, bilateral trade volumes, and direct flights) that are of great concern to the drivers of New Zealand-inbound business tourism (Tsui et al., 2018). In this premise, two groups of thoughts exist in the context of the correlation of tourism inflow and outflow, and business confidence index: one believing that there is no direct relationship, while the others being of the opinion that there is a direct relationship between the two constructs. In the study of several researchers (Bodo et al., 2000; Gholipour & Foroughi, 2020; Taylor & McNabb, 2007), they believe that there is a positive connection between tourism and business confidence index, ascertaining that when there is confidence about business opportunities and growth in the international trade, then there will be an increase in the inflow of business people to that area or country. They concluded that when there is a perception that there is a low level of business confidence in a particular country, this will thus create adverse effect on the economy and will reduce travel demand to such country (Khan & Upadhayaya, 2020; Milani, 2017). Specifically, Khan and Upadhayaya (2020) used quarterly data for the case of the USA over a period of 60 years to determine the information on business confidence for investors. The study presents three positive results. At first, the finding suggests that business confidence has the ability to determine growth. On the other hand, it also suggests that business confidence enhances the predictive power of investors. Lastly, the findings witness exogenous shifts in business confidence. This is to say that the confidence of conducting business is expected to be positive. But there is another argument that opined that there is no correlation between tourism and business confidence. Their augments are based on the fact that having confidence on business without depending on the macro-economic indicators will yield no result, thus suggesting a relation between business confidence and tourism (Mourougane & Roma, 2003; Santero & Westerlund, 1996).

### Consumer Leading Indicators and Tourism Aspects

In recent days, the relationship between tourism activities and consumer confidence has drawn great attention making it a point of interest for policymakers (Juhro & Iyke, 2020). The study of business tourism demand is very vital, as it stands as a yardstick to evaluate consumer’s response in case of any economic impediments. Several studies are of the opinion that consumer confidence is related to tourism activities (Poudyal et al., 2013; Turner & Witt, 2001). Some economists assumed that the global financial crisis is a result of the lack of confidence on the economy (Dees & Brinca, 2013). They argue that during the period of uncertainty in having an insight into consumer consumption behaviour, consumer expectations are the major key variables (Eugenio-Martin & Campos-Soria, 2014).

A very recent study by Hampson et al. (2021), on how price-conscious behaviour affects consumers using a structural equation modelling technique, was implemented with data collected from 1090 US consumers. Differentiating the consumers into national consumer confidence and personal consumer confidence, the study drew from the attribution theory and found out that consumer confidence has an influence on national consumer confidence and higher on consumers with a high external locus of control. Again, Ghosh (2021) utilized a nonlinear autoregressive distributed lag model to examine the stock market behaviour and financial stress in relation to consumer confidence to policy uncertainty using a seasonally adjusted monthly variable from 1995 to 2018. The result confirmed that there is asymmetric impact of policy uncertainty in Japan, that is to say that the impact of a fall in asymmetry on consumer confidence is lesser than the rise in policy uncertainty.

Although financial crisis cannot be attributed to consumer sentiments alone, other economic indicators are also relatable (Gholipour & Tajaddini, 2018). Gholipour and Tajaddini (2018) found a relation between the subdimensions of consumer confidence and tourist outgoing expenditures across 22 European countries using a panel data regression. The result shows a positive association between tourist expenditure and consumer confidence. From the result, it was ascertained that tourists with a high level of confidence in the future spend more than tourists with low levels of confidence. Interestingly, more recent scholars have found that significant relationship exists between tourism activities and economic policy uncertainty (EPU) (Akron et al., 2020; Akadiri et al., 2020; Liu et al., 2021; Wu & Wu, 2021; Hailemariam & Ivanovski, 2021a, 2021b). These studies ascertained that tourism has a significant relationship with EPU. Looking from another dimension of primary data source, Voorchees et al. (2021) employed 3084 customers for 2 years with primary research to examine the effects of service variability on consumer confidence. Their findings show that variability in services can influence consumer confidence; this is to say that any change in quality will have an effect on consumer confidence. On the other hand, their study suggests that firms can encourage consumers to engage in relational investments which increase consumer confidence. Additionally, Gholipour et al. (2021) propounded that there is a relative relation between consumer confidence and changes in economic policy. They examined this using data from 2005 to 2019 with six countries and found that a positive change in uncertainty will result in a negative effect on tourist departure.

### Study Relevance and Hypotheses

The array of the above-mentioned studies, although limited in the literature, illustrates the link between the aspects of tourism market-related confidence indicators (such as business and consumer confidence). However, what was not clearly depicted in the literature, which now remains unknown, can be identified in different ways. First, there is limited evidence to see if market confidence displays a distinct role in determining inbound and outbound tourism. Secondly, none of the aforementioned studies provides relevant information about the existence or otherwise of asymmetric relationship between market confidence indicators and tourism aspects. Lastly, of course, there is existing information about Granger causality among market confidence indicators and tourism aspects. However, this study employs the recent approach of Granger causality with inference of frequency domain which provides additional contribution in this study. Thus, relevant hypotheses based on the gap in the literature and limited discussion are contextualized from the following relationships:

- The linear or symmetric short- and long-run relationship between market confidence indicator and tourism inflow and outflow.
- The nonlinear or asymmetric short- and long-run relationship between market confidence indicator and tourism inflow and outflow.
- The frequency domain Granger causality between market confidence indicator and tourism inflow and outflow.

## Dataset and Method

The variables employed for the study include the number of tourists visit to Russia (hereby named as Trussia) and outbound tourism from Russia (hereby named as Frussia) which were all retrieved from the Federal State Statistic Service database (Federal State Statistic Service, 2021). Alongside the aforementioned variables which are the dependent variables, the business confidence index (hereby named as BCI) and consumer leading indicator index (hereby named as CLI) are the main (explanatory) variables, while the consumer price index (hereby named as CPI) is utilized to control for the unspecified factors. These explanatory variables were retrieved from the Economic Research database of the Federal Reserve Bank of St. Louis (2021). The dataset is spanned over the quarterly period 2010Q1–2020Q4.

Moreover, the statistical properties of the concerned variables are illustrated in Table 1. In general, the variables, except for consumer leading indicators, are all normally distributed and (except for tourist visits to Russia) negatively skewed. Considering the downturn of economic activities as brought about by the coronavirus (COVID-19) pandemic situation that triggered lockdown and restriction of movement policies across the world, especially in 2020, the tourist visits to Russia recorded a minimum value of 0 as observed in Table 1. Similarly, a minimum value of 109 persons reported travelled out of Russia. This lower value is also a reflection of the restriction in movement of people due to the traumatic period of the COVID-19 pandemic.

**Table 1:** Descriptive statistics of the variables

Variable	FRUSSIA	TRUSSIA	BCI	CLI	CPI
Mean	3,325,073	670,996.0	100.814	100.120	95.854
Median	3,165,936	509,665.0	100.910	100.093	100.054
Maximum	6,090,450	2,144,987	102.363	103.573	125.167
Minimum	109.000	0.000	98.821	93.833	64.456
Standard dev	1,418,067	518,217.2	0.832	1.717	19.928
Skewness	− 0.012	0.971	− 0.275	− 0.706	− 0.117
Kurtosis	2.667	3.302	2.407	5.878	1.496
Jarque–Bera	0.205	7.075	1.197	18.841	4.2478
Probability	0.903	0.029	0.550	0.000^e^	0.120
Observations	44	44	44	44	44

(1) While ^e^ indicates the 1% statistically significant level, Standard dev. implies the standard deviation. (2) FRUSSIA, TRUSSIA, BCI, CLI, and CPI are variables that, respectively, describe tourist outflow from Russia, tourist inflow to Russia, business confidence index, consumer confidence index, and consumer price index

### Model and Preliminary Estimations

Considering that this study is designed to look at the changes in tourist visits to Russia and outbound tourism from Russia along the determining factors of BCI, CLI, and CPI, the following two models are implied:

1 $${\text{LFRussia}} = f\left( {{\text{BCI}},{\text{CLI}},{\text{CPI}}} \right),$$

2 $${\text{LTRussia}} = f\left( {{\text{BCI}},{\text{CLI}},{\text{CPI}}} \right),$$

where the values of Frussia and Trussia are transformed into logarithmic form as indicated by L.

Following the illustrated statistical inference in Table 1, other preliminary estimations that include the stationarity, cointegration, and the nonlinearity tests were essentially performed. The stationarity estimates as conducted with the Lee and Strazicich (2003) unit root approach revealed that the variables are stationary at most after the first difference with evidence of break dates (see Table 6 of appendix). The break dates were largely reported between the third-quarters of 2012 and 2019. These periods account for important events associated with the Russia Federation, which includes the era marking the beginning of Vladimir Putin’s second presidency term, height of Ukraine and Russia skirmish, and the Russia involvement in Syria, which is against the Western countries’ and United Nations’ perception. Moreover, the cointegration evidence provided by the approach of Johansen and Juselius (1990) as revealed in Table 7 of the appendix presents a statistically significance evidence of at most two cointegrating equation in the illustrated models. Following the evidence of long-term association among the experimented variables, the Broock et al (1996) approach was adopted to provide inference for a nonlinearity relationship (see Table 2), thus offering the path to investigate the nonlinear impacts of the BCI, CLI, and CPI on both TRussia and FRussia.

**Table 2:** The nonlinearity preliminary test

Variables	BDS statistic	*Z*-statistic	Probability value
LFRussia	− 0.143	− 6.575	0.000^e^
LTRussia	− 0.143	− 6.576	0.000^e^
BCI	0.068	8.748	0.000^e^
CLI	0.119	8.405	0.000^e^
CPI	0.202	32.170	0.000^e^

(1) ^e^ is the 1% statistically significant level. (2) L represents logarithmic transformation

### Method

Given that the null hypothesis of linear relationship is rejected as provided in Table 2, the study proceeds with the application of the nonlinear approach by Shin et al (2014). The Shin et al (2014) approach was a modification or incorporation of the nonlinearity parameters to the earlier linear dimension of autoregressive distributed lag (ARDL) approach by Pesaran et al (2001). Thus, applying the nonlinear dimension of the ARDL (NARDL) of Shin et al (2014) to models (equation) 1 and 2, the respective nonlinear expression becomes

3 $${\text{LFRussia}}_{t} = C_{{{\text{LFRussia}}}} + C_{1}^{ + } BCI_{t}^{ + } + C_{2}^{ - } BCI_{t}^{ - } + C_{3}^{ + } CLI_{t}^{ + } + C_{4}^{ + } CLI_{t}^{ - } + C_{5}^{ + } CPI_{t}^{ + } + C_{6}^{ + } CPI_{t}^{ - } + {\mathfrak{e}}_{t} ,$$

4 $${\text{LTRussia}}_{t} = C_{{{\text{LTRussia}}}} + C_{1}^{ + } BCI_{t}^{ + } + C_{2}^{ - } BCI_{t}^{ - } + C_{3}^{ + } CLI_{t}^{ + } + C_{4}^{ + } CLI_{t}^{ - } + C_{5}^{ + } CPI_{t}^{ + } + C_{6}^{ + } CPI_{t}^{ - } + {\mathfrak{e}}_{t} ,$$

where BCI, CLI, and CPI exert both positive and the negative impacts on LFRussia and LTRussia, with dimensions indicated as the respective coefficients. The respective intercept of the estimation is indicated as ${C}_{\mathrm{LFRussia}}$ and ${C}_{\mathrm{LTRussia}},$ while the error term is ${\mathfrak{e}}_{t}$ over the indicated period *t*: ${t}_{1}$ = 2010Q1, ${t}_{2}$ = 2010Q2,…, ${t}_{44}$ = 2020Q4.

Now, we apply the step-by-step procedure to Eq. (3) and the same procedure is replicated for Eq. (4). Indicatively, the partial sum of BCI, CLI, and CPI (all explanatory variables denoted as A) is decomposed into negative and positive as follows:

5 $$A_{t}^{ + } = \sum\limits_{i = 1}^{t} {\Delta A_{i}^{ + } } = \sum\limits_{i = 1}^{t} {\max (\Delta A_{i} ,0)} ,\quad {\text{while}}\quad A_{t}^{ - } = \sum\limits_{i = 1}^{t} {\Delta A_{i}^{ - } } = \sum\limits_{i = 1}^{t} {\max (\Delta A_{i} ,0).}$$

Thus, the nonlinear dimension of the ARDL takes the form

6 $$\begin{aligned} {\text{LFRussia}}_{t} & = C_{0} + C_{{{\text{LFRussia}}}} {\text{LFRussia}}_{t - 1} + C_{1}^{ + } BCI_{t - 1}^{ + } + C_{2}^{ - } BCI_{t - 1}^{ - } + C_{3}^{ + } CLI_{t - 1}^{ + } + C_{4}^{ + } CLI_{t - 1}^{ - } + C_{5}^{ + } CPI_{t - 1}^{ + } + C_{6}^{ + } CPI_{t - 1}^{ - } \\ & + \sum\limits_{i = 0}^{p - 1} {B_{{{\text{LFRussia}}}} \Delta {\text{LFRussia}}_{t - 1} } + \sum\limits_{i = 0}^{q - 1} {B_{1} \Delta BCI_{t - 1}^{ + } } + \sum\limits_{i = 0}^{q - 1} {B_{2} \Delta BCI_{t - 1}^{ - } } \\ & + \sum\limits_{i = 0}^{q - 1} {B_{3} \Delta CLI_{t - 1}^{ + } } + \sum\limits_{i = 0}^{q - 1} {B_{4} \Delta CLI_{t - 1}^{ - } } + \sum\limits_{i = 0}^{q - 1} {B_{5} \Delta CPI_{t - 1}^{ + } } + \sum\limits_{i = 0}^{q - 1} {B_{6} \Delta CPI_{(t - 1)}^{ - } } + {\mathfrak{e}}_{t} . \\ \end{aligned}$$

While the above-expressed equations present the combination of the ordinary least square and long-run relationship between the explanatory (mostly of positive and negative shocks) with lag order *q* and dependent variables with lag order *p*, the coefficients represent the respective impacts with the error term${\mathfrak{e}}_{t}$. Moreover, the short- and long-run asymmetry are tested by applying the F- and t-statistic under the null hypothesis of no cointegration, respectively, by B = B^+^ = B^−^ = 0 and C = C^+^ = C^−^ = 0. While ${B}_{i}^{+}$ and ${B}_{i}^{-}$ directly measure the positive and negative impacts in the short run, the long-run dimensions are measured by ${A}_{i}^{+}$= $\frac{{C}^{+}}{{C}_{\mathrm{LFRussia}}}$ and = ${A}_{i}^{-}\frac{{C}^{-}}{{C}_{\mathrm{LFRussia}}}$, where A denotes the set of explanatory variables. This procedure is also replicated for the second model, i.e. tourist visits to Russia.

#### Robustness Tests

In providing a robustness evidence to the result provided by the NARDL estimate, a combination of Granger causality and linear cointegration approaches was employed. Specifically, the more recent Granger causality approach that offers frequency causative inference by Breitung and Candelon (2006) and Pesaran et al (2001) that provides linear short- and long-run relationship was employed. Although the stepwise procedures are not illustrated in this study because of space constraint, the details are covered in the related literature.

## Discussion of Findings

The results of the nonlinear relationship between the examined set of variables are implied in Table 3 for the two investigated models, i.e. LTRussia and LTFRussia. In the case of the trend of the tourist visit to Russia, both negative and positive shock in business confidence exerts a significant and positive effect in the short and long run. Although a negative shock in business confidence is shown to exert larger impact on tourist visit to Russia, the revelation from the result implies that confidence in business activities within Russia would not necessarily affect the positive attitude of tourists to visit Russia. This inference is expected, since a larger percentage of the visitors to Russia are from European neighbours, who have according to the Federal State Statistic Service (2021) a relatively stronger exchange rate advantage, and thus changes in the business activities in the destination countries might be immaterial. More so, the same perception is likely to hold for the relationship between the consumer price index and tourist visit to Russia as also indicated in the outlined result. However, a shock (either negative or positive) in the consumer leading indicator triggers a statistically significant impact that yields a decline in the number of tourist visits to Russia, and largely in the two periods (short and long run). Although the study of Yap and Allen (2011) found that both business confidence and consumer sentiments exert certain impact on some aspects of domestic tourism such as visit to friends and relatives, the current study reveals that a shock on consumer confidence about future economic developments could demotivate inbound tourism to Russia. The negative observation in the aforementioned relationship could be attributed to the response of potential tourists to some of the components of the consumer leading indicators or to one of the overbearing indicators.

**Table 3:** Short- and long-run nonlinear estimates

Coefficient	Inbound tourism to Russia (LTRussia)		Outbound tourism from Russia (LFRussia)	
	Coefficient	Probability value	Coefficient	Probability value
Long run				
BCI^positive^	0.159	0.049^f^	0.036	0.910
BCI^Negative^	0.256	0.006^e^	0.817	0.003^e^
CLI^Posotive^	− 0.126	0.001^e^	− 0.149	0.279
CLI^Negative^	− 0.146	0.036^f^	− 0.574	0.002^e^
CPI^Positive^	0.001	0.911	− 0.002	0.958
CPI^Negative^	0.000	0.001^e^	0.000	0.700
Short run				
BCI^positive^	− 0.250	0.049^f^	0.256	0.599
BCI^Negative^	0.603	0.001^e^	1.830	0.007^e^
CLI^Posotive^	− 0.297	0.070^g^	− 0.853	0.034^f^
CLI^Negative^	0.250	0.054^g^	2.017	0.000^e^
CPI^Positive^	0.002	0.958	0.222	0.061^g^
CPI^Negative^	0.000	0.900	0.000	0.900
Diagnostic estimates				
*F*-statistic (probability value > *F*) =	136.79 (0.000)		41.00 (0.000)	
*R*-squared (adjusted *R*-squared) =	0.991 (0.984)		0.967 (0.943)	
Heteroskedasticity test (probability value) =	0.163 (0.686)		3.107 (0.078)	
RESET test (probability value)	= 2.581 (0.085)		49.67 (0.000)	
Normal test with Jarque–Bera statistics	= normally distributed			

RESET regression specification error test

^e,f,g^ is 1%, 5%, and 10% statistically significant levels, respectively

However, for the outbound tourism from Russia, a shock (mostly negative) in the consumer confidence reduces the number of Russian travellers to a tourist destination outside the country in the short- and long-term period. This observation is expected because an unexpected turn of event that robs the confidence of Russians, especially from the perspective of the future economic developments, is capable of discouraging potential outbound tourism intention. The conclusion from this result is also similar to that of the study of Dragouni et al (2016), where sentiment and mood are revealed to transmit spillover shocks to potential outbound tourism activities in the case of the USA. As regards the business confidence and consumer price index, a statistically significant value is only observed when there is a negative shock on the business confidence. Thus, the result revealed that a negative shock in business confidence prompts more touristic travels by Russians to destination countries, while shock in the consumer price index does not yield a meaningful implication.

**Fig. 1:**
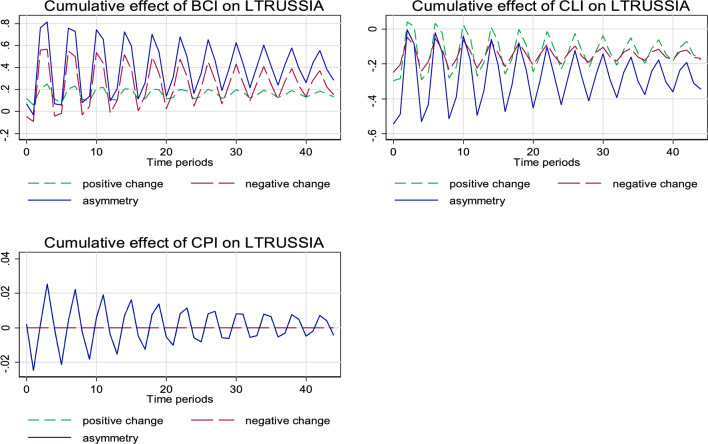
The cumulative effect graph for the model ‘Tourists’ visit to Russia (LTRussia)’

**Fig. 2:**
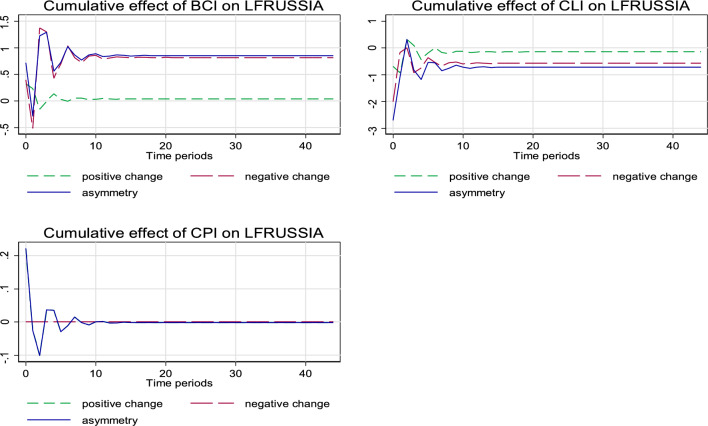
The cumulative effect graph for the model ‘Outbound tourism from Russia (LFRussia)’

In general, the short- and long-run asymmetric estimates as illustrated in Table 4 further confirms an asymmetric relationship that is mostly positive. Adding to the desirable diagnostic inference from the models’ R-squared, F-statistic, and normality as indicated in the lower part of Table 3, Figs. 1 and 2 present more diagnostic observation for the estimated models. Evidently from Fig. 1, both the positive and negative shocks in BCI and CLI exert statistically significant asymmetric effect on inbound tourism to Russia, while no statistical significance is observed for the asymmetrical relationship between CPI and inbound tourism to Russia. For the outbound tourism, as illustrated in Fig. 2, the case is similar to the inbound tourism scenario as described above. However, in this case, the negative asymmetric effect is stronger for both the relationship of BCI and CLI with outbound tourism from Russia.

**Table 4:** Asymmetric estimates

	Inbound tourism to Russia (LTRussia)		Outbound tourism from Russia (LFRussia)	
	*F*-statistic	Probability value	*F*-statistic	Probability value
Asymmetric long run				
BCI	37.28	0.000^e^	7.734	0.010^f^
CLI	11.25	0.003^e^	21.03	0.000^e^
CPI	0.013	0.911	0.003	0.958
Asymmetric short run				
BCI	10.3	0.004^e^	0.891	0.355
CLI	4.47	0.047^e^	20.96	0.000^e^
CPI	0.214	0.648	3.458	0.075^g^

RESET regression specification error test

^e,f,g^ is 1%, 5%, and 10% statistically significant levels, respectively

### Robustness

To provide robustness, the application of the linear ARDL method provides the result of the relationships in Table 5. The result implies that there is a quarterly adjustment of about 35% to secure an equilibrium stability, especially during a short-run to long-run transition. For the long-run estimate, there is statistically significant evidence that business confidence hinders both inbound and outbound tourism, while the impacts of consumer leading indicator and consumer price index are statistically significant and positive. Moreover, the short-run observation is not outrightly different, because business confidence also exerts a statistically significant impact on both inbound and outbound tourism, while consumer leading indicator and consumer price index are positively related with inbound and outbound tourism.

**Table 5:** Symmetric estimates

	Inbound tourism to Russia (LTRussia)		Outbound tourism from Russia (LFRussia)	
	Coefficient	Probability value	Coefficient	Probability value
Symmetric long run				
BCI	− 2.804	0.043^f^	− 1.910	0.089^ g^
CLI	2.660	0.005^e^	1.385	0.017^f^
CPI	0.095	0.058^ g^	0.048	0.183
Symmetric short run				
ECM	− 0.349	0.005^e^	− 0.367	0.000^e^
BCI	− 0.978	0.011^f^	− 0.701	0.013^f^
CLI	2.576	0.000^e^	1.655	0.000^e^
CLI (-1)	− 1.108	0.000^e^	− 1.147	0.000^e^
CPI	0.192	0.86	0.018	0.074^g^
Bound test				
*F*-statistic (*k* = 3) = 4.103 where 5% critical values for *I* (0) = 2.79 and *I* (1) = 3.67				
Stability evidence by cumulative and cumulative of squares tests indicate that the models are stable				

RESET regression specification error test

^e,f,g^ is 1%, 5%, and 10% statistically significant levels, respectively

**Fig. 3:**
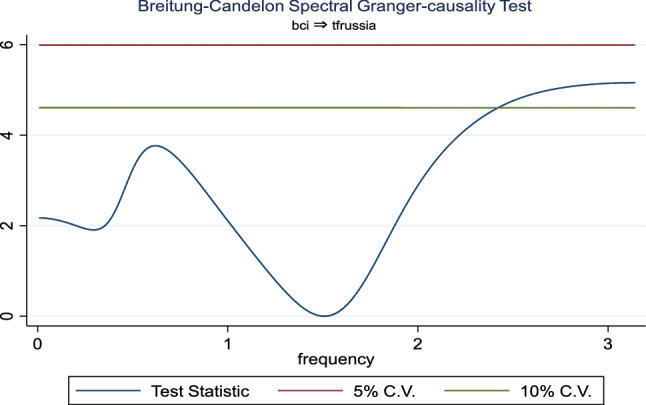
Granger causality from business confidence to outbound tourism from Russia

**Fig. 4:**
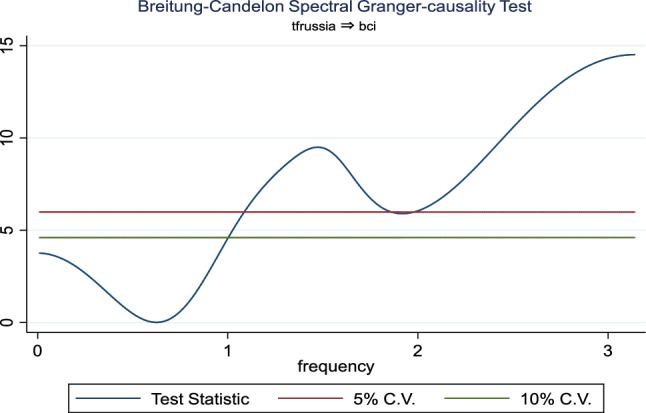
Granger causality from outbound tourism from Russia to business confidence

**Fig. 5:**
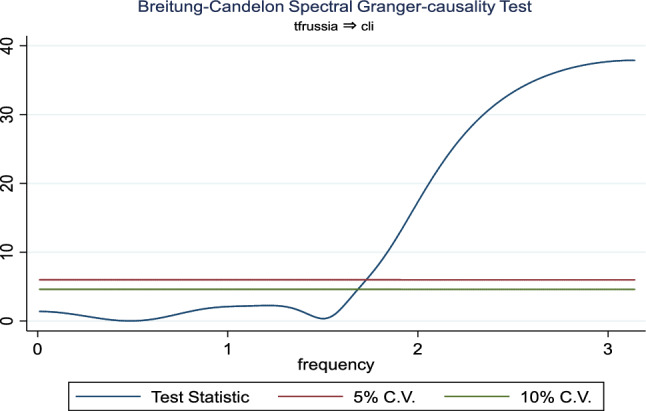
Granger causality from outbound tourism from Russia to consumer leading indicator

**Fig. 6:**
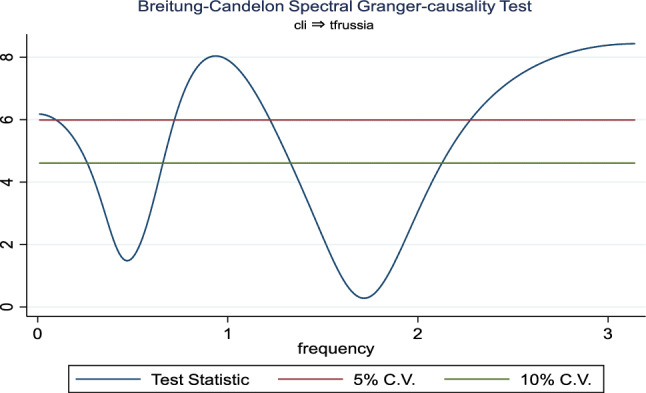
Granger causality from consumer leading indicator to outbound tourism from Russia

**Fig. 7:**
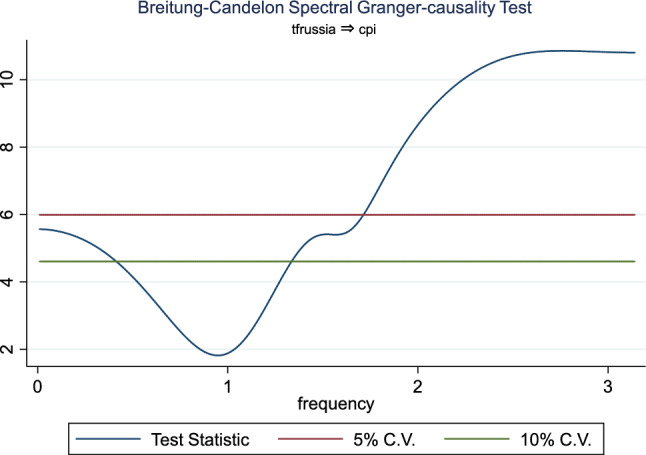
Granger causality from outbound tourism from Russia to consumer price index

**Fig. 8:**
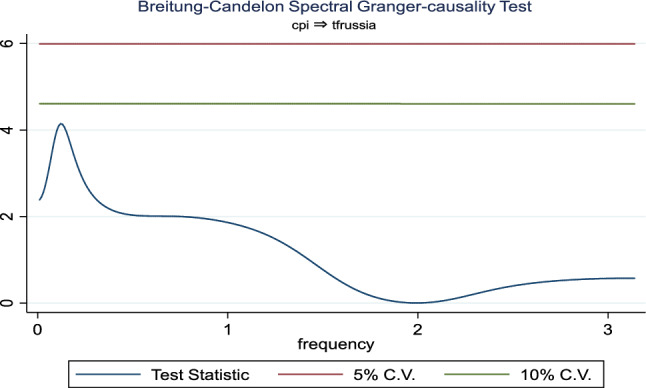
Granger causality from consumer price index to outbound tourism from Russia

Additionally, the frequency domain Granger causality evidence between the examined explanatory variables and outbound tourism is provided in Figs. 3, 4, 5, 6, 7, and 8. The inbound tourism was not used because zero observation was recorded in 2020Q2, thus making the estimation technique incompatible. However, the result revealed a causality between BCI and outbound tourism, especially a bidirectional relationship in the medium term. Similarly, the CLI and outbound tourism Granger causality is prevalent in the medium term as a two-way relationship, while the Granger causality from CPI to outbound tourism is a one-way relationship that is persistent in the short and medium term.

## Conclusion and Policy-Related Dimension

Before 2019, prior to the coronavirus pandemic that has disrupted the global economic activities, the Russia Federation has long benefited from the significant contribution from the country’s tourism sector. Moreover, the outbound tourism from Russia is more about a historical rite for many Russians, especially for the desire to experience a relatively tropical climate. In this context, the current study examined the determinants of inbound tourism and outbound tourism from Russia from the perspective of business confidence, consumer indicator vis-à-vis consumer leading indicator, and consumer price index for the quarterly period 2010Q1–2020Q4. We performed the symmetric and asymmetric analysis of the relationship by using the linear and nonlinear ARDL approach while also examining the Granger causality from the dimension of frequency domain causation. Therefore, the investigation revealed some significant findings.

- Findings revealed that negative and positive shock in business confidence exerts positive impact on inbound tourism in the short and long run by a statistically significant amount. But a negative shock in business confidence shows a larger effect. This observation is also affirmed for the relationship between the consumer price index and tourist visit to Russia.
- A significant decline in the number of tourist visits to Russia in both short and long run arises from the impact of the shock (irrespective of the direction) in the consumer leading indicator.
- A shock (mostly negative) in the consumer confidence reduces the number of Russian travellers to foreign tourist destinations in the short- and long-term period. However, when there is a negative shock on the business confidence, it causes more Russian travellers to embark on foreign touristic destination, while the consumer price index shows no significant effect.
- As for the linear relationships, business confidence hinders both inbound and outbound tourism, while consumer leading indicator and consumer price index exert positive effect. Although there is not much difference in the impact of business confidence in the short run, the consumer leading indicator and consumer price index are also positively related with inbound and outbound tourism in the short run.
- Moreover, the frequency domain Granger causality evidence reveals Granger causality (mostly bidirectional) from BCI to outbound tourism, and from CLI to outbound tourism mostly in the medium term. However, the Granger causality from CPI to outbound tourism is a one-way relationship that is persistent in the short and medium term.

### Policy Issues

The direction of the policy implication is much in line with the measures to boast the attraction of the tourism industry in Russia. Thus, it is important that business activities in the country are geared towards supporting inbound tourism through the implementation of business-friendly policy such that it attacks investment in the tourism sector. For instance, moderate or specialized interest rate regime for the tourism sector could encourage more investment in the sector and to a large extent business activity, thus promoting competitiveness among business actors. Such competitiveness in the industry is capable of lowering the cost of goods and services, which are key determinants of tourists’ intention, thus promoting inbound tourism to Russia. The other aspects of economic sector that promote tourism in Russia such as education, sports (example is skiing), and traditional tourism should be driven with appropriate policy. Another dimension of policy could be assessed from the perspective of the determinants of consumer confidence. To improve consumer confidence such that tourism activities are expanded in the country, there should be improvement in the welfare benefits for citizens such as unemployment claims, employee wages, and purchasing manager index. Moreover, a better welfare package is expected to increase the economic status, thus encouraging more Russians to embark on either domestic or outbound tourism for reasons of leisure, medical, and other reasons.

For future purpose, this study could follow another approach. For instance, the role of these examined factors (business–consumer confidence, consumer price index, and others) could be examined from the perspective of tourism purpose(s). This will provide more relevant information to the specific link between the examined variables and the sector-related specific purposes.

### Key Questions Reflecting Applicability in Real Life

Notably, curious questions that could prompt relevant immediate and future opportunities in the sector and geared towards economic development includes.

1. How does the country identify key market forces that define the framing of the new competitive tourism market model?
2. What are the practical approaches that should be taken to improve the long-term market image and industry competitiveness?
3. What are the immediate policy measures that could further spur tourism inflow from Asian and European tourism, the two closest tourism markets?
4. How can the country’s private sector participation in tourism be scaled up in the direction of improving the industry’s competitiveness?

## Data Availability

The dataset is freely available online as indicated in the data description section.

## Appendix

See Tables

**Table 6:** Stationarity test with break dates

	Level tau	First difference tau	Date
BCI	− 5.724	− 6.983^e^	2012Q3 2019Q1
CLI	− 4.853	− 7.538^e^	2012Q3 2018Q1
CPI	− 7.892^e^	− 8.297^e^	2014Q3 2016Q3
LFRussia	− 11.112^e^	− 13.228^e^	2015Q2 2019Q3
LTRussia	− 10.396^e^	− 6.077^g^	2013Q2 2016Q4

^e,f,g^ is 1%, 5%, and 10% statistically significant levels, respectively. tau is the minimum test statistic

6 and

**Table 7:** Evidence of cointegration in the models

Model: LFRUSSIA = *f* (BCI, CLI, CPI)				
Trace test				
# of cointegrating eq	Eigen value	Trace statistic	Critical value	Probability value
None	0.484	58.268	47.856	0.004^e^
At most 1	0.414	30.463	29.797	0.042^f^
At most 2	0.168	8.039	15.495	0.461

Maximum eigenvalue test				
# of cointegrating eq	Eigenvalue	Max Eigen statistic	Critical value	Probability value
None	0.484	27.804	27.584	0.047^f^
At most 1	0.414	22.424	21.132	0.033^f^
At most 2	0.168	7.731	14.264	0.407

Model: LTRUSSIA = *f* (BCI, CLI, CPI)				
Trace test				
# of cointegrating eq	Eigenvalue	Trace statistic	Critical value	Probability value
None	0.951	153.227	47.856	0.000^e^
At most 1	0.397	35.701	29.797	0.009^e^
At most 2	0.318	15.955	15.495	0.043^f^

Maximum eigenvalue test				
# of cointegrating eq	Eigenvalue	Max Eigen statistic	Critical value	Probability value
None	0.951	117.526	27.584	0.000^e^
At most 1	0.397	19.746	21.132	0.077^g^
At most 2	0.318	14.944	14.265	0.039^f^

^e,f,g^ is 1%, 5%, and 10% statistically significant levels, respectively

7.
